# Activating the Antitumor Immune Response in Non-Hodgkin Lymphoma Using Immune Checkpoint Inhibitors

**DOI:** 10.1155/2020/8820377

**Published:** 2020-11-18

**Authors:** Maansi Joshi, Stephen M. Ansell

**Affiliations:** Division of Hematology, Mayo Clinic, Rochester, MN, USA

## Abstract

Non-Hodgkin lymphomas comprise a heterogenous group of disorders which differ in biology. Although response rates are high in some groups, relapsed disease can be difficult to treat, and newer approaches are needed for this patient population. It is increasingly apparent that the immune system plays a significant role in the propagation and survival of malignant cells. Immune checkpoint blocking agents augment cytotoxic activity of the adaptive and innate immune systems and enhance tumor cell killing. Anti-PD-1 and anti-CTLA-4 antibodies have been tested as both single agents and combination therapy. Although success rates with anti-PD-1 antibodies are high in patients with Hodgkin lymphoma, the results are yet to be replicated in those with non-Hodgkin lymphomas. Some lymphoma histologies, such as primary mediastinal B cell lymphoma (PMBL), central nervous system, and testicular lymphomas and gray zone lymphoma, respond favorably to PD-1 blockade, but the response rates in most lymphoma subtypes are low. Other agents including those targeting the adaptive immune system such as TIM-3, TIGIT, and BTLA and innate immune system such as CD47 and KIR are therefore in trials to test alternative ways to activate the immune system. Patient selection based on tumor biology is likely to be a determining factor in treatment response in patients, and further research exploring optimal patient populations, newer targets, and combination therapy as well as identifying biomarkers is needed.

## 1. Introduction

Immune therapies have changed the paradigm of cancer treatment, particularly Hodgkin and non-Hodgkin lymphomas. Lymphoma cells, being a part of the immune system, are themselves immunologically active and modulate the host immune response to allow growth of the malignant cell. In addition, the tumor microenvironment (TME) is now being increasingly recognized for its role in immune suppression and propagating tumor growth. Interactions between lymphoma cells and the TME influence T cell function are crucial for tumor progression. Checkpoint proteins act as natural regulators of T cell function and help to modulate the T cell response by creating a balance between activation and inhibition [1].

Cytotoxic T lymphocyte antigen 4 (CTLA-4/CD152) and programmed cell death protein 1 (PD-1/CD279) of the B7 family, among others, are inhibitory molecules which result in reduced T cell activity and function. Disease tolerance seen in malignancy can be attributed in part to sustained interaction of these proteins with their corresponding ligands on antigen presenting cells (APCs) [2]. Monoclonal proteins targeting immune checkpoints such as anti-CTLA-4 antibodies and anti-PD-1 and anti-PD-1 ligand (PD-L1 and PD-L2) antibodies have shown promising results in the treatment of solid tumors and hematological malignancies. This review will discuss the role of these antibodies as well as other immune checkpoint inhibitors (CPI) in non-Hodgkin lymphoma (NHL).

## 2. Role of Tumor Microenvironment in Immune Escape

**Figure 1:**
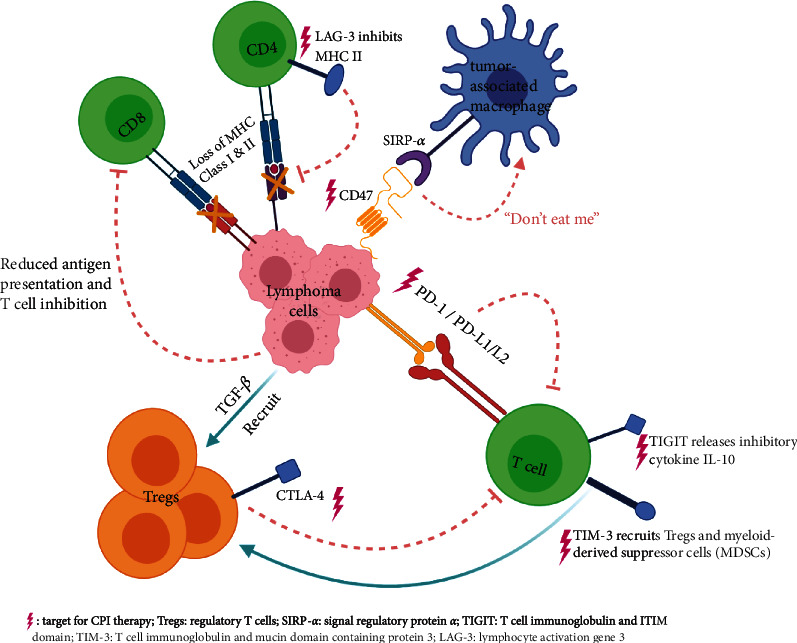
Mechanisms of immune escape by lymphoma cells.

Malignant B cells in lymphoma have the ability to evade host immune responses, and this is in part due to lymphoma cell interactions with the tumor microenvironment (TME) (Figure 1). The TME is complex and heterogenous and comprises of tumor cells, immune cells, stromal cells, blood vessels, and a variety of associated tissue cells. Immune cells present in the tumor include components of the innate (macrophages, dendritic cells, etc.) and adaptive immune system (B and T cells). T cell activation, which is the first step in mounting an effective immune response, occurs when antigen presenting cells (APCs) such as macrophages and dendritic cells present foreign antigens to host T cells. Activation of T cells is initiated via T cell receptor engagement with major histocompatibility complex (MHC) class I and II molecules on APCs. A second activating signal, typically mediated via CD28, CD27, and tumor necrosis factor receptor superfamily proteins, is required for adequate T cell function. An overenthusiastic T cell response is mitigated by induction of T cell inhibitory signals via CTLA-4, PD-1, CD160, and B and T lymphocyte-associated protein (BTLA) [3]. Tumor cells capitalize on these regulatory pathways by overexpressing inhibitory ligands or secreting immunosuppressive cytokines, thereby dampening an effective immune response [4].

An effective and appropriate immune response relies on adequate antigen presentation in the context of MHC molecules. Lymphoma cells themselves act as antigen presenting cells but are only weakly immunogenic because of reduced expression of MHC on their surface [5]. Loss of MHC occurs either due to homozygous deletion of MHC class II genes or chromosomal translocations in the MHC master regulator [6, 7], resulting in reduced presentation of tumor-associated antigens to host CD4+ T helper cells and therefore reduced activation of cytotoxic T lymphocytes (CTLs). These findings have been confirmed by DNA microarray analysis that shows fewer CTLs in the TME in MHC II negative than MHC II expressing DLBCL biopsy samples [8, 9]. The role of MHC II can be further affected by lymphocyte activating gene 3 (LAG-3) signaling, an inhibitory coreceptor that binds to MHC class II additionally suppressing its function [10]. Furthermore, MHC loss triggers upregulation of CD47 which interacts with signal regulatory protein alpha (SIRP*α*) to send a “don't eat me” signal to phagocytic cells within the TME [11].

Overexpression of inhibitory ligands on lymphoma cells can suppress an effective antitumor T cell response. Genetic amplification in lymphoma cells at the chromosome 9p locus and associated upregulation of the JAK2 genes results in expression of aberrant surface markers, particularly CD274 and CD273 or programmed cell death ligands 1 and 2 (PD-L1/PD-L2). These proteins interact with the PD-1 receptor on CD4+ T cells and CTLs to provide inhibitory signals as a negative regulator of T cell activity. Uncontrolled immune activation has been documented in PD-1 and PD-L1 null mice, highlighting the importance of balance between stimulatory and inhibitory signals in T cell function [12, 13]. In lymphoma patients, however, there may be upregulation of the PD-L1 and/or PD-L2 expression on the malignant cells that inhibits the T cell response and results in a state of reduced T cell differentiation, decreased proliferation, and suppressed effector function. Upregulation of these ligands is also seen within the TME cells, commonly on intratumoral macrophages. Immune checkpoints in lymphoma may be amenable to therapeutic manipulation to allow T cell reactivation either by blocking inhibitory interactions or promoting agonistic stimulatory signals.

Additionally, regulatory T cells (Tregs) that are part of the TME suppress T cell function and play an important role in immune homeostasis and self-tolerance by inhibiting cytotoxic T cells. Lymphoma cells actively recruit Tregs to the TME to suppress the antitumor immune response and promote Treg differentiation via immunosuppressive cytokines such as tumor growth factor *β* (TGF-*β*). TGF-*β* suppresses effector T cell function by causing T cells to differentiate into Tregs [14]. Tregs express CTLA-4 which is an inhibitory molecule that suppresses T cell function by assisting in Treg-mediated downregulation of stimulatory molecules CD80 and CD86 on dendritic cells. Inhibition of CTLA-4 inhibits FoxP3+ Tregs from inhibiting effector T cell function [15]. Due to signaling induced by inhibitory ligands, the secretion of immunosuppressive cytokines, and the presence of cells with regulatory function, the antitumor immune response is effectively suppressed in most lymphoma patients.

## 3. Biological Basis of Checkpoint Blockade in NHL

As mentioned above, T cell function is governed by stimulatory and inhibitory molecules to ensure an optimal response. Immune checkpoint proteins, which tend to be upregulated in lymphoma, send inhibitory signals to activated T cells and cause suppression of T cell function. Reactivating T cells forms the basis of immune checkpoint inhibitor (CPI) therapy. An ongoing understanding of T cell biology has prompted the development of therapies capable of restoring T cell function. As a strategy to prevent T cell suppression, checkpoint inhibitors have been developed against multiple inhibitory proteins responsible for regulating the adaptive immune system including PD-1/PD-L1, CTLA-4, TIM3, LAG3, and TIGIT and against inhibitory pathways regulating the innate immune system including CD47. Here, we discuss the rationale behind some of these therapies.

### 3.1. PD-1/PD-1 Ligand Signaling

The therapeutic success of immune checkpoint blockade in classical Hodgkin lymphoma (cHL) has largely been attributed to the high prevalence of 9p24.1 amplification and the enhanced PD-1/PD-L1/2 expression on tumor cells and in the TME in this disease. In contrast, NHLs are more heterogenous and do not share the same biological features as cHL, in that the PD1/PD-L1 expression in NHL has inconsistently been associated with prognosis [16]. However, there are subgroups of NHLs that share a common genetic signature, and high rates of 9p24.1 gain similar to cHL. These include primary mediastinal B cell lymphoma (PMBL), primary CNS lymphoma (PCNSL), primary testicular lymphoma (PTL), and gray zone lymphoma (GZL) [17–21], all of which have increased expression of PD-L1/2 due to genetic upregulation. Relative expression of PD-L2, compared to PD-L1, is increased in PMBL with more than 70% of tumor cells expressing the ligand and polymerase chain reaction (PCR) testing confirming copy number gains in PD-L2 in these patients [17]. In patients with PCNSL and PTL, recurrent translocations of regulatory elements of *TBLX1XR1* and PD-L2 gene as well as genes upstream of PD-L1 and PD-L2 were observed [18]. These tumors, in comparison to other types of NHL, have high expression of PD1, PD-L1, and/or PD-L2, which forms the basis for CPI in these conditions, and therapeutic responses have been seen in these diseases in early phase clinical trials.

In contrast, 9p24.1 copy number alterations (CNAs) resulting in the increased PD-1/PD-L1 expression are seen in only a handful of patients with de novo diffuse large B cell lymphoma (DLBCL), and the link to prognosis in these patients is not clear [22]. Burkitt lymphoma and mantle cell lymphoma cells also have virtually no PD-L1/2 expression detected on tumor cells [16, 23]. Similarly, follicular lymphoma cells seldom have 9p24.1 gain and have inconsistent expression of PD-L1 on their surface. However, the high PD-1 expression on tumor infiltrating lymphocytes (TILs) of the TME in follicular lymphoma (FL) has been observed, and this has variably corresponded with a shorter time to progression or high-grade transformation [16, 24–27]. PD-1 and LAG-3 signaling blockade resulted in CD8+ T cell function restoration in these patients [28]. In comparison, DLBCL has lower levels of PD-1 expressing TILs, and patients with DLBCL have been noted to have circulating PD-1, but the prognostic impact of these findings remains unclear [29–32]. Whether the expression of checkpoint proteins on cells from the TME in lymphoma predicts sensitivity to CPI therapy is as yet unknown.

Chronic viral infection, especially with EBV, upregulates the PD-1 [23] and PD-L1 [33] expression, and the PD-L1 expression corresponds with clinical responses to PD-1 blockade [16]. This provides a rationale for the use of CPI in EBV-associated lymphomas such as EBV positive DLBCL, NK/T cell lymphomas, and posttransplant lymphoproliferative disorders (PTLD). PTLD in particular show high levels of both PD-1 and PD-L1 expression and may be amenable to CPI therapy.

### 3.2. CTLA4 Expression

CTLA-4 is an inhibitory receptor of the B7 family and negative regulator of T cell activation. It can be detected on regulatory T cells (Tregs), as well as activated CD4+ and CD8+ T cells, and acts by enhancing Treg function and dampening the immune response [15]. CTLA-4 blockade has been shown to enhance the activity of endogenous antitumor T cells, thereby inducing tumor regression [34]. Anti-CTLA-4 antibodies, ipilimumab and tremelimumab, have been extensively studied in melanoma patients, and in 2011, the Federal Drug Administration (FDA) approved ipilimumab for treatment of metastatic melanoma. In patients with NHL, the efficacy of CTLA4 inhibition was modest at best, with only 2 of 18 patients showing a response in a phase 1 trial [35]. However, combination therapies that include anti-CTLA-4 antibodies and synergistic agents to prime the immune system have significantly enhanced antitumor responses, as shown in the EL4 lymphoma mouse model where coadministration of dendritic cell vaccine and anti-CTLA-4 antibody resulted in tumor responses in 60% of the mice, whereas neither agent alone prevented tumor growth [36]. This suggests that the efficacy of CTLA4 inhibition may require additional modulation of immune function.

### 3.3. Other Checkpoint Proteins

In addition to the two checkpoint molecules mentioned above, inhibitory molecules against other immune checkpoint proteins are currently being investigated. These proteins include T cell immunoglobulin and ITIM domain (TIGIT), T cell immunoglobulin and mucin domain containing protein 3 (TIM-3), and lymphocyte activation gene 3 (LAG-3). TIGIT interacts with the poliovirus receptor (PVR) and increases IL-10 production, which is an inhibitory cytokine [37, 38]. Anti-TIGIT antibodies have shown some promising results in mouse models [39], and clinical trials are being designed to test them further. TIM-3 regulates T helper cells and helps with recruitment of myeloid-derived suppressor cells (MDSCs), which are potent suppressors of T cell immunity via production of nitric oxide (monocytic MDSCs) and pathways involving IFN-*γ*, production of reactive oxygen species, and arginine metabolism (granulocytic MDSCs) [40]. High expression of TIM-3 on DLBCL tumor cells was associated with a worse overall and progression free survival [41]. Trials using anti-TIM-3 antibodies in relapsed lymphoma are ongoing to test safety and efficacy in human subjects. Similarly, LAG-3 interacts with CD3 to mediate inhibition of T cell proliferation and cytokine production [42]. Anti-LAG-3 antibodies are also being tested in hematological malignancies.

### 3.4. Targeting Regulators of the Innate Immune System

Macrophages, monocytes, and natural killer (NK) cells form part of the innate immune system and a similarly controlled by immune regulatory receptors. CD47, a regulator of the phagocytosis, is ubiquitous on all normal tissues with upregulation seen in malignant cells. The receptor for this ligand is signal regulatory protein *α* (SIRP*α*), which is present on monocytes and macrophages, dendritic cells, and granulocytes. Interaction between these proteins sends a “do not-eat-me” signal to macrophages and monocytes which downregulates malignant cell phagocytosis [11, 43]. Anti-CD47 antibodies can block this inhibitory signal and promote phagocytosis, particularly antibody-mediated cellular cytotoxicity (ADCC) [44]. However, blockade of this pathway has the potential of off-target toxicity due to the universal expression of CD47 [45] that includes anemia and thrombocytopenia. Despite this, humanized anti-CD47 antibody molecules have been shown to promote macrophage-mediated phagocytosis in NHL engrafted mouse xenografts without indiscriminate killing of normal cells [46, 47]. This has translated into clinical benefit with a phase Ib trial of anti-CD47 antibody in combination with rituximab showing a 40% response rate in DLBCL and 71% response rate in FL [48].

Another potential therapeutic target that may regulate the innate immune system is the killer-cell immunoglobulin-like receptor (KIR) expressed on NK cells. Interaction between KIR and MHC class I molecules induces NK cell tolerance [49]. Anti-KIR antibodies have shown enhancement of NK cell-mediated cytotoxicity, an effect further augmented when combined with rituximab [50].

### 3.5. Combination Therapy

Single agent CPI results in responses in only a fraction of patients with non-Hodgkin lymphoma. Combination therapies using strategies that further enhance immune function are currently being explored and include improving antigen presentation, promoting T helper cell response, dual checkpoint blockade, and enhancing T cell activation. Chemotherapy and radiotherapy, which disrupt DNA, enhance expression of pattern recognition receptors (PRRs) on cells of the innate immune system thereby improving antigen presentation by APCs and macrophages [51, 52]. This forms a basis for current trials investigating combination chemotherapy or radiotherapy with CPI in lymphomas.

Aside from the immune checkpoint combinations mentioned above, small molecule inhibitors have potential activity in combination with CPI. One agent which has shown synergy *in vitro* is ibrutinib, a BTK inhibitor which suppresses B cell receptor (BCR) signaling that is essential for malignant B cell survival. Ibrutinib, however, also targets interleukin-2 inducible kinase (ITK) in T cells and may shift the balance between Th1 and Th2 T cells, thereby enhancing the antitumor response. Lymphoma mouse models have shown that ibrutinib can potentiate T cell responses in presence of PD-1 blockade causing a synergistic effect [53].

Further, mouse models show where PD-1 and TIM-3 coexpressing TILs are present; they represent a more exhausted phenotype and dual blockade of TIM-3 and PD-1 may result in better tumor control [54]. Similar results were seen in an A20 lymphoma mouse model which showed coexpression of PD-1 and TIGIT, and near complete disappearance of tumor was seen when mice were treated with both anti-PD-1 and anti-TIGIT antibodies [55]. In a further mouse model, the combination of an anti-PD-1 antibody with an anti-CTLA-4 antibody resulted in an augmented antitumor response due to an increase in intratumoral cytokines [56]. These preclinical results suggest that combinations of immune checkpoint blocking antibodies may result in a more effective antilymphoma T cell response than the use of each CPI alone. These combinations may also include immune checkpoint inhibitors targeting both the adaptive and innate immune system. Clinical results thus far testing these combinations have been disappointing. In a phase 1 study combining PD-1 blockade (nivolumab) with either an anti-CTLA-4 antibody (ipilimumab) or anti-KIR antibody (lirilumab) resulted in an ORR of 9-22% in patients with NHL, and toxicity from the combinations was greater than that seen with the single agents alone [57]. Other clinical studies to establish the most effective combinations are currently ongoing.

## 4. Clinical Efficacy of Checkpoint Inhibition

Despite the promise of preclinical studies, the clinical efficacy of CPI in NHL has been modest as shown by a number of early phase clinical trials. In some subclasses of NHL, the response rates are as high as 40%-50%, particularly in patients with a 9p24.1 amplification that results in PD-L1/2 overexpression. In other types of lymphoma, the response rates are poor. Unfortunately, a large percentage of patients with NHLs do not gain benefit from CPI therapy. Currently, there are 20 registered trials studying efficacy of checkpoint blockade as single agents or in combination with other therapies in patients with different types of NHL. A summary of select completed trials is listed in Table 1.

### 4.1. NHL with Upregulation of 9p24.1

Some subgroups of non-Hodgkin lymphoma which show amplification of the 9p24.1 locus respond very well to PD-1 blockade. These histologies include primary mediastinal B cell lymphoma (PMBL), gray zone lymphoma (GZL), primary CNS lymphoma (PCNSL), and primary testicular lymphoma (PTL). The KEYNOTE-170 trial, which enrolled 53 patients with relapsed/refractory PMBL, reported an ORR of 45% with a 13% CR rate in pembrolizumab-treated patients. At a median follow-up of 12.5 months, the median duration of response and overall survival was not reached. At the time of the report, all patients in CR continued in CR including one patient off therapy for 12 months [58, 59]. Similar promising results have been observed in patients with relapsed/refractory PCNSL and PTL treated with nivolumab. In a small series of patients, all five patients (4 PCNSL and 1 PTL) responded including 4 CRs after 3 cycles of treatment. At a follow-up of 17 months, three maintained a response, and all 5 patients were alive [60]. In addition, 3 of 3 patients with gray zone lymphoma in a separate report achieved durable CR with PD-1 blockade [61].

### 4.2. Diffuse Large B Cell Lymphoma

Disappointingly, checkpoint inhibitors as single agent therapy have limited efficacy in diffuse large B cell lymphoma (DLBCL). In a phase 1 trial of ipilimumab, an anti-CTLA-4 antibody, in patients with relapsed or refractory NHL, only one patient with DLBCL had a durable response [35]. Results with PD-1 blockade were initially more promising. In a phase 1 study of nivolumab, an ORR of 35% was observed among patients with DLBCL; however, durability was poor with most patients relapsing by 3 months [62]. Subsequently, in the CHECKMATE-139 trial, nivolumab was administered to 121 patients with relapsed/refractory DLBCL (r/r DLBCL) who had either failed or were ineligible for an autologous stem cell transplant (ASCT). At a median posttreatment follow-up of 9 months in the auto-ASCT–failed cohort and 6 months in the auto-ASCT–ineligible cohort, response rates were 10% and 3%, respectively. The median durations of response were less than 12 months with a median OS 12.2 months and 5.8 months, respectively. Further, only 3% of patients showed evidence of chromosome 9p24.1 amplification [63]. Similarly, pembrolizumab was studied in patients with DLBCL as consolidation post-ASCT with the goal of improving the PFS at 18 months from 60% to 80%. Unfortunately, in the 29 patients enrolled, the 18.5-month PFS was 58% with increased toxicity confirming posttransplant PD-1 blockade does not improve therapeutic benefit in unselected DLBCL patients.

One subgroup of large cell lymphoma that has shown some initial encouraging results is patients with Richter's transformation (RT). These patients tend to have the high PD-1 expression on the tumor cells [64], and a promising response rate to pembrolizumab therapy was demonstrated in a cohort of patients with CLL [65]. Confirmatory studies were unfortunately disappointing with most patients in the KEYNOTE-170 RT cohort progressing. Only 3 (one with DLBCL histology and 2 with cHL histology) of 23 patients responded and even those patients had poor response durability [66].

In contrast, checkpoint inhibitors targeting proteins of the innate immune system appear to have greater efficacy. Blockade of CD47/SIRP*α* signaling with TTI-621 showed an ORR of 36% and CR of 14% in r/r DLBCL [67]. Treatment with Hu5F9-G4, an anti-CD47 antibody, plus rituximab resulted in an ORR of 40% with a CR rate of 33% in DLBCL patients [48].

Similar to single agent therapy, combination immune checkpoint therapy in unselected DLBCL patients has shown limited efficacy. Nivolumab combined with ipilimumab demonstrated an ORR of 36% in r/r DLBCL with increased toxicity compared to single agent [68]. Further, nivolumab combined with either ipilimumab or lirilumab (anti-KIR antibody) resulted in an ORR of 19% and 13%, respectively, in patients with NHL in a phase 1 trial [57]. Similarly, and despite *in vitro* efficacy, ibrutinib combined with PD-1 blockade resulted in an ORR of 36% only in a clinical study [69]. In select populations, however, the results are more encouraging. Thirty patients with r/r PMBL treated with nivolumab and brentuximab vedotin in a phase 2 trial showed an ORR of 70% with CR of 43% [70]. These data stress the importance of appropriate patient selection in patients with lymphomas, and studies combining tumor-directed therapy and immune checkpoint blockade in selected populations are now underway.

### 4.3. Follicular and Other Low-Grade Lymphomas

Relapsed/refractory low-grade lymphomas can be challenging to treat due to their propensity to relapse. Although thought to be inherently immunosensitive due to response to nonspecific immune blockade, results with CPI in follicular lymphoma (FL) have been mixed. An initial phase 1 study with ipilimumab showed only a single durable response [35]. More promising results were seen with nivolumab in a phase I trial showing a 40% response rate, and most responses were durable for up to 2 years [62]. However, CHECKMATE-140, a phase 2 trial of nivolumab in relapsed/refractory FL (r/r FL), showed very modest results in the enrolled 92 patients with an ORR of only 4%, a median DOR of 11 months, and a PFS of only 2.2 months [71]. Success with pembrolizumab has also been limited, as seen in a phase 2 trial of low-grade lymphomas which included 23 patients: 18 with FL, 2 with marginal zone lymphoma (MZL), and 3 with lymphoplasmacytic lymphoma (LPL). Two FL patients had partial responses, and one LPL patient had a minor response, but the duration of response (DOR) was only 5.5 and 4.9 months, respectively, and the MZL patient having a 40% tumor reduction with ongoing therapy. Overall, the median PFS for all patients was only 3.4 months [72].

Combination therapy in FL has been more encouraging in comparison. In a phase 1 trial of atezolizumab and obinutuzumab, the ORR was 57% in r/r FL [73], whereas a phase 2 trial of pembrolizumab with rituximab demonstrated an ORR of 80% with a CR rate of 60%. The median PFS and OS in this study were not reached at 7 months [74]. Further, rituximab combined with utomilumab achieved an ORR of 33% in patients with r/r FL who were rituximab refractory [75]. Ongoing clinical trials are now exploring combination therapies with checkpoint blockade and chemoimmunotherapy, radiotherapy, and immunomodulators.

### 4.4. T Cell Lymphoma

T cell lymphomas are typically a difficult group of diseases to manage, and these diseases are often associated with a poor prognosis especially in the relapsed/refractory setting. Response to CPI therapy is often dictated by the type of T cell lymphoma with some histologies responding better than others. NK/T cell lymphomas, which are often associated with EBV infection, have been shown to have an upregulation of the PD-L1 expression and tend to respond favorably to PD-1 blockade [76, 77]. In the r/r setting, 5 of 7 patients in one cohort and 7 of 7 patients in a second cohort responded to PD-1 blockade. Other types of T cell lymphomas have had more mixed results. Nivolumab in r/r PTCL and mycosis fungoides showed an ORR of 40% and 15%, respectively, in a phase 1 trial [62]. A phase 2 trial of pembrolizumab enrolled 23 patients with advanced MF. The ORR was 38% with 89% of responses durable at a median of 32 weeks resulting in a one-year PFS of 69%. In contrast, only one of 13 patients with PTCL achieved a response with combination therapy with nivolumab and ipilimumab [68]. Despite some studies showing reassuring results, there have been reports of hyperprogression in some cohorts on patients with PTCL. At least 2 groups of investigators have reported rapid disease progression in a subset of patients after initiation of checkpoint inhibition [78, 79].

### 4.5. Summary

Responses seen with checkpoint inhibitor therapy in NHL do not compare with the success seen in cHL. Some patients with NHL respond well to this therapy, especially those with chromosome 9p24.1 amplification, including patients with PMBL, GZL, PCNSL, and PTL. A further group of responding patients is those with EBV positive lymphomas which also tend to upregulate the PD-1/PD-L1 expression [80, 81]. Specifically, NK/T cell lymphomas which tend to be EBV driven and also show upregulation of PD-L1 have demonstrated encouraging results. Other EBV positive lymphomas such as posttransplant lymphoproliferative disorder (PTLD) which have upregulated the PD-L1 expression may also be amenable to checkpoint blockade [81].

## 5. Adverse Events with Checkpoint Blockade

As discussed, checkpoint blockade inhibits the immunosuppressive T cell signal which allows tumor cells to evade the immune system and proliferate undetected. As a result, there is immune activation when these agents are administered therapeutically. Although the exact mechanism is unknown, the working hypothesis is that most side effects are due to failure of immunological tolerance resulting in T cell-mediated immune-related adverse events (irAE) [82]. Furthermore, cytokines may also play a role as evidenced by a rise in interleukin-17 levels in patients with ipilimumab-induced colitis [83]. These events usually occur within the first few weeks or months after therapy is initiated. Delayed onset of immune side effects after therapy cessation has also been reported with waxing and waning of symptoms. Encouragingly, data from melanoma studies does not show any long-term side effects [84]. Fatal toxic events have been reported and occurred in 0.3% to 1.3% of patients treated, usually occurring early in the treatment course [85]. Immune-related AEs can affect almost any organ and may be different depending on the immune checkpoint inhibitor used, e.g., colitis and hypophysitis are more common with anti-CTLA-4 therapy, and pneumonitis and thyroiditis are more common with anti-PD-1 therapy. Overall, immune adverse events appear to be more common with CTLA-4 blockade (60%-90%) compared to PD-1/PD-L1 blockade (40%-70%), with the incidence being higher in patients receiving combination therapy [82, 86]. Myocarditis is a rare side effect of CPI, and in hematological malignancies treated with CPI, it is almost never seen. The exact reason for this discrepancy is as yet unknown.

Many events can be managed with simple measures such as the use of anti-inflammatory or antipyretic agents including NSAIDs or antihistamines, or by stopping the offending drug. Sometimes, medications that can curb an overactive T cell response are necessary such as corticosteroids, calcineurin inhibitors, and anti-TNF*α* inhibitors like infliximab. Whether it is safe to restart CPI therapy after an irAE has occurred has not been studied in prospective trials. Retrospective analysis suggests that recurrent or new immune reactions can occur with repeat therapy [87]. While these events tend to be less severe, the decision to recommence treatment should be individualized depending on nature of the malignancy, the severity of the prior event, and whether feasible alternative treatment options are available.

A concerning feature reported in some T cell lymphoma trials with CPI therapy is rapid disease progression resulting in death. Twelve patients with relapsed/refractory PTCL (r/r PTCL) were treated with nivolumab at the Mayo Clinic, and although 4 patients responded to treatment, there were 4 instances of hyperprogression, defined as dramatic disease progression within one cycle of treatment. The study was terminated early as a result [79]. Furthermore, 3 patients with adult T cell leukemia lymphoma (ATLL) treated with nivolumab showed rapid disease progression after one dose of the medication [78]. In addition, Zinzani et al. [88] noted 3 episodes of hyperprogression leading to death in a cohort of 44 patients with r/r PTCL treated with Tislelizumab [88]. Careful thought therefore needs to be given to patient selection in T cell lymphoma trials utilizing CPI therapy.

## 6. Biomarkers

Checkpoint therapy has shown some remarkable results in early phase trials; however, not all patients respond to treatment and novel biomarkers to predict response and help patient selection are therefore necessary. We know from studies in solid tumors that the increased PD-1/PD-L1 expression is associated with responses to checkpoint blockade [89, 90]. In patients with lymphoma, however, the predictive value of the PD-1/PD-L1 expression is inconsistent. Trials in Hodgkin lymphoma showed that the elevated PD-L1 expression on tumor cells corresponded with a higher ORR [91, 92]. *H* scores, calculated as the number of PD-L1 positive malignant cells multiplied by the intensity of PD-L1 positive staining, correlated with 9p24.1 amplification, which in turn predicted response. However, some patients with low *H* scores also had a clinical response [93].

In contrast to Hodgkin lymphoma, the usefulness of the PD-L1 expression, detected in the TME or serum, as a biomarker in DLBCL has been less clear. Soluble PD-L1 (sPD-L1) was detected in pretreatment plasma samples from an initial cohort of 283 DLBCL patients from France and a confirmatory cohort of 225 patients from North America. Both cohorts showed an inferior overall survival in patients with elevated sPD-L1. This however did not correlate with tumor or TME expression of PD-L1, and tumor PD-L1 did not predict survival in patients [94, 95]. In a study using nivolumab in relapsed DLBCL, the prevalence of copy number gain or amplification of chromosome 9p24.1 was low, and the tumor expression of PD-L1 did not correlate with response. Of the 2 patients who had a CR, one had 9p24.1 amplification, whereas the other one had normal levels. In those with PR, none had the PD-L1 expression on tumor cells [63].

However, in a small cohort of patients with Richter's transformation treated with pembrolizumab, the increased PD-L1 expression was detected in patients with a confirmed response. Other markers tested included chromosome 9p24.1 alterations, EBV status, and MSI status, but none of these correlated with treatment response in CLL patients with RT [65]. In the KEYNOTE-170 trial of pembrolizumab in relapsed PMBL, the tumor *H* score was calculated for PD-L1. A rise in *H* score signifying higher expression of PD-L1 and amplification of 9p24.1 gain strongly correlated with response to therapy, and the PD-L1 expression was also strongly associated with progression free survival [59].

In a study of patients with follicular lymphoma, the higher expression of PD-L1 was seen at baseline on peripheral blood T cells among responders when treated with pidilizumab [96]. Further, the increased expression of a T cell activation gene signature in pretreatment tumor samples was associated with a longer PFS in patients treated with PD-1 blockade. This was not seen in a separate study where patients were treated with chemotherapy alone [96]. This suggests that the gene signature may be useful in predicting outcomes in patients post PD-1 blockade therapy, but larger studies with higher patient numbers are needed to confirm this finding.

## 7. Conclusion

Checkpoint inhibitors disrupt the inhibitory feedback loop of the immune system rather than target tumor cells. Treatment with these agents has shown promising results in some relapsed and refractory non-Hodgkin lymphomas, which continue to be an area of unmet need. However, appropriate patient selection appears to be the key in determining treatment outcome, as some histologies of NHL respond well to CPI while many others do not. As discussed in this review, patients with primary mediastinal B cell lymphoma, primary CNS lymphoma, testicular lymphoma, and gray zone lymphoma are most likely to respond favorably. The PD-L1 expression has been shown to predict response in some studies, but this is inconsistent, and a reliable biomarker to predict response is currently lacking. Moving forward, newer ICP targets will need to be considered. Potential molecules including TIM-3, LAG-3, TIGIT, and BTLA have shown encouraging results in preclinical models, and clinical trials exploring their efficacy are currently accruing patients. Agents targeting the innate immune system are also being investigated, and anti-CD47 and anti-KIR antibodies appear to hold promise in early studies. Furthermore, combination therapy with more than one CPI or CPI with other targeted agents and/or chemotherapy is being investigated. All told, we anticipate that rational combination approaches that include immunological agents will be necessary to improve the outcome of patients with NHL.

## Figures and Tables

**Table 1:** Select completed trials of immune checkpoint blockade in non-Hodgkin lymphoma.

Reference	Phase	Disease	No. of patients	Drug	Results
Anti-CTLA-4 antibody					
Ansell et al., 2009 [35]	I	rrFL, rrDLBCL	18	Ipilimumab	ORR 11%; 1 CR DLBCL; 1 PR FL
Anti-PD-1 antibody					
Armand et al., 2019 [59]	II (KEYNOTE-170)	rrPMBL	53	Pembrolizumab	ORR 45%, CR 13%; mDOR NR
Ding et al., 2017 [65]	II	rrCLL, RT	RT = 9 CLL = 16	Pembrolizumab	ORR 44%, CR 11%, mPFS 5.4 months. No response in CLL
Ding et al., 2017 [72]	II	rr LPL, FL, MZL	23	Pembrolizumab	ORR 17.4%, mPFS 3.4 months
Khodadoust et al., 2016 [97]	II	rrMF + Sezary syndrome	24	Pembrolizumab	ORR 38%, CR 8%, 1 year PFS 69%
Ansell et al., 2019 [63]	II (CHECKMATE-139)	rr DLBCL	121	Nivolumab	ORR 10% (failed ASCT), mPFS 1.9, mOS 12.2 months; ORR 3% (ineligible ASCT), mPFS 1.4 months, OS 5.8 months
Nayak et al., 2017 [60]		rr PCNSL, PTL	PCNSL = 4 PTL = 1	Nivolumab	ORR 100%, CR 80%; 3 patients remain in CR at 13-17 months
Armand et al., 2013 [98]	II	DLBCL consolidation post auto	66	Pidilizumab	ORR 51%, mPFS at 16 months 72%
Combination therapy					
Ansell et al., 2016 [68]	I (CHECKMATE-039)	NHL	BNHL = 15 TNHL = 11	Nivolumab + ipilimumab	B NHL: ORR 20%, PR 20%, mOS 2.9 months T NHL: ORR 9%, PR 9%, mOS 13.2 months
Palomba et al., 2017 [73]	Ib	rrDLBCL, rrFL	49	Atezolizumab (anti-PD-1) + obinutuzumab	ORR 57% for FL and 16% for DLBCL
Nastoupil et al., 2017 [74]	II	rrFL	27	Pembrolizumab + rituximab	ORR 80%, CR rate 60%. Median DOR, PFS, and OS NR at 7 months
Advani et al., 2018 [99]	Ib/2	rr NHL	DLBCL = 15 FL = 7	Hu5F9 (anti-CD47) + rituximab	DLBCL: ORR 40%, CR 27% FL: ORR 71%, CR 43%

## Data Availability

Not required.
